# Supplementary material for: Risk factors influencing Coxiella burnetii seropositivity in water Buffalo (Bubalus bubalis) populations of Egypt’s Nile Delta
Source: PLoS One. 2025 Oct 7;20(10):e0333680. doi: 10.1371/journal.pone.0333680 (PMC12503306; doi:10.1371/journal.pone.0333680)
Supplement: Attachment — Submitted filename: Review report.pdf [file pone.0333680.s001.pdf]

The manuscript presents an important investigation on C. burnetii in water buffalo which has an important public health significance; however, several improvements in structure, clarity, and language are necessary to enhance its scientific value and reproducibility.

Abstract

- Line 23-24: Please rewrite "Buffaloes over 8 years of age showed a higher prevalence (21.6%) compared to the younger age groups"

Materials and methods

- Line 93: Authors quoted "conducted using a convenient sample of cattle, selected through simple random sampling from herds…". Did they mean a convenient sample of the herds and a random sampling of animals within herds? Please explain how this was actually done to make the study reproducible.
- Line 99-100: A more details of sample collection is required, volume of each sample collected in which type of container (e.g., EDTA or clot activator etc.), who collected the samples, was the samples taken in ice box immediately, and a time range within which the samples were processed in lab for centrifugation, and how long the samples were stored in -20°C before serological analysis?
- Line 128-134: The Authors did not address confounding, interaction, and collinearity for the final regression model. If any confounding is found that variable must be included in the final model to correct for the confounding effect. If there is collinearity, only one variable should be chosen to eliminate the duplication of effects.
- For me, this is actually a multivariable regression, not multivariate, since the outcome is binary (yes/no)

Results

- Line 413 Table 2: Please keep the reference category for each variable in the table to make the comparison easily understandable. Please improve the table captions e.g., Multivariable logistic regression analysis for *C. burnetii* regressed against four variables as risk factors from N=(440) buffaloes in four governorates in Egypt's Nile Delta
- Line 415: CI: 95% Confidence intervals
- Results of confounding and collinearity check should be mentioned in results

Discussion

- My suggestions are that the discussion may start with a brief paragraph on the important findings, especially the higher prevalence and risk factors how it could be important for the perspective of the study population. This partly done in line 165-167 where author can specify the risk factors e.g., older buffaloes, communal grazing area, presence of tick infestation, small ruminants, and previous history of abortion.
- Line 171 "Seroprevalence rate" rate should be carefully interpreted, rate usually is relevant to incidence, which is different from prevalence.
- Could the ELISA be a limitation for example, antibody may not always represent actual presence of the pathogen and since the Se and Sp of ELISA might be lower than PCR? The authors may also report this, which could result in a minor underestimation or overestimation of actual prevalence.
- Line 184-188: Authors may explain the source of variations e.g., "Environmental factors, including climate and geography, also play a role in disease transmission." How the transmission occurs in response to 1 or 2 of those important variables.
- "discrepancies in diagnostic tools, testing protocols, sample sizes, and study designs can lead to differing results " Similarly here, authors may explain here 1 or 2 of those important variables. That will make the discussion more informative.
- Line 204-207 "Additionally, vaginal discharges, …… animals during communal grazing [43]." Does it mean that in communal grazing, the management seems more difficult than in non-grazing?
