# Supplementary material for: Risk factors influencing Coxiella burnetii seropositivity in water Buffalo (Bubalus bubalis) populations of Egypt’s Nile Delta
Source: PLoS One. 2025 Oct 7;20(10):e0333680. doi: 10.1371/journal.pone.0333680 (PMC12503306; doi:10.1371/journal.pone.0333680)
Supplement: Attachment — Submitted filename: Response to reviewers comments.doc [file pone.0333680.s005.doc]

Journal Requirements:

**Point#:** If the reviewer comments include a recommendation to cite specific previously published works, please review and evaluate these publications to determine whether they are relevant and should be cited. There is no requirement to cite these works unless the editor has indicated otherwise.

Response#: the references were reviewed
**Point#:** Please review your reference list to ensure that it is complete and correct. If you have cited papers that have been retracted, please include the rationale for doing so in the manuscript text, or remove these references and replace them with relevant current references. Any changes to the reference list should be mentioned in the rebuttal letter that accompanies your revised manuscript. If you need to cite a retracted article, indicate the article’s retracted status in the References list and also include a citation and full reference for the retraction notice.
Response#: the references list was reviewed and corrected
Additional Editor Comments:

**Point#:** I personally reviewed the latest revisions submitted by the authors (due to the unavailability of reviewer #1). The authors did not submit a "track changes manuscript" (the two uploaded files are identical). Furthermore, some comments were not addressed. I ask the authors to address all reviewer comments and reflect these changes in the manuscript, marking them with the track changes tag.

**Response#:** comments of reviewer 1 were addressed by red color
