# Supplementary material for: Risk factors influencing Coxiella burnetii seropositivity in water Buffalo (Bubalus bubalis) populations of Egypt’s Nile Delta
Source: PLoS One. 2025 Oct 7;20(10):e0333680. doi: 10.1371/journal.pone.0333680 (PMC12503306; doi:10.1371/journal.pone.0333680)
Supplement: Attachment — Submitted filename: Response to reviewer comments.doc [file pone.0333680.s004.doc]

**Comments to the Author**

1. If the authors have adequately addressed your comments raised in a previous round of review and you feel that this manuscript is now acceptable for publication, you may indicate that here to bypass the “Comments to the Author” section, enter your conflict of interest statement in the “Confidential to Editor” section, and submit your "Accept" recommendation.

Reviewer #1: (No Response)

Reviewer #3: All comments have been addressed

2. Is the manuscript technically sound, and do the data support the conclusions?

The manuscript must describe a technically sound piece of scientific research with data that supports the conclusions. Experiments must have been conducted rigorously, with appropriate controls, replication, and sample sizes. The conclusions must be drawn appropriately based on the data presented.

Reviewer #1: Partly

Reviewer #3: Yes

3. Has the statistical analysis been performed appropriately and rigorously?

Reviewer #1: I Don't Know

Reviewer #3: Yes

4. Have the authors made all data underlying the findings in their manuscript fully available?

The [PLOS Data policy](http://track.editorialmanager.com/CL0/http:%2F%2Fwww.plosone.org%2Fstatic%2Fpolicies.action%23sharing/1/010f0198ec2e7353-6b4376c0-e176-455c-8ca9-fc5c00655f02-000000/qmMmS1Uhn1zJZp00O_sEUTDAG6E7Vx03s2bTO5tG70w=226) requires authors to make all data underlying the findings described in their manuscript fully available without restriction, with rare exception (please refer to the Data Availability Statement in the manuscript PDF file). The data should be provided as part of the manuscript or its supporting information, or deposited to a public repository. For example, in addition to summary statistics, the data points behind means, medians and variance measures should be available. If there are restrictions on publicly sharing data—e.g. participant privacy or use of data from a third party—those must be specified.

Reviewer #1: No

Reviewer #3: Yes

5. Is the manuscript presented in an intelligible fashion and written in standard English?

PLOS ONE does not copyedit accepted manuscripts, so the language in submitted articles must be clear, correct, and unambiguous. Any typographical or grammatical errors should be corrected at revision, so please note any specific errors here.

Reviewer #1: Yes

Reviewer #3: Yes

**Review Comments to the Author**
Reviewer #1: A number of comments from the previous round were not addressed. Please see them below.
**Point#:** Lines 93 and 100: This states that cattle were sampled and blood was collected from cattle, respectively. Shouldn’t this be from buffalo?

Response#: it corrected
**Point#:** Line 101 states cattle were sampled.

Response#: it corrected
**Point#:** Line 127: Please include how confounders were handled.
As described in statistical analysis, potential confounders such as sex, age, communal grazing practices, contact with small ruminants, presence of tick infestation, and history of abortion were considered. Initially, these variables were examined using univariable analysis. Factors with *p* < 0.20 in the univariable screening were subsequently included in a multivariable logistic regression model to adjust for possible confounding effects. The final model retained variables that remained significant at *p* < 0.05.
**Point#:** Lines 138 – 151: Please include clarity throughout this section so the reader knows this is based on univariable analysis. Similarly, please clarify in the title of Table 1 as well.
Response#: it rephrased
**Point#:** Lines 190-195: Suggest removing this section since the findings were not at the significance threshold set by the authors.
Response#: it removed
**Point#:** Lines 193 and 229: Please include a reference to support the statements that pregnant females have greater susceptibility to C. burnetii infection.

Response#: reference 46
**Point#:** Line 238: The statement that female sex is significantly associated with infection does not align with the results section or Tables.

Response#: line 238 discussed that the seroprevalence increased significantly in animals with history of abortion, not discussed the sex
**Point#:** Table 1:
- The > before 5-8 should be removed. Same for Table 2. Same for line 106.

Response#: it removed

- How is tick infestation defined? Please clarify somewhere in the manuscript. Is it the presence of any ticks or > a certain number? Is the status determined at the time of blood collection?

Response#: it defined as presence on the coat of animals at time of collection and clarified in materials and methods
- yes in the “History of abortion in previous year” row is lowercase.
Response#: it corrected
**Point#:** Line 101: please clarify what pf stands for.

Response#: it corrected

Reviewer #3:

No further comments on the updated version. Thanks to the authors for sufficiently revising the manuscript.

Thank you for your valuable comments and your time
